# Cost-Related Medication Nonadherence and Desire for Medication Cost Information Among Adults Aged 65 Years and Older in the US in 2022

**DOI:** 10.1001/jamanetworkopen.2023.14211

**Published:** 2023-05-18

**Authors:** Stacie B. Dusetzina, Robert J. Besaw, Christine C. Whitmore, T. Joseph Mattingly, Anna D. Sinaiko, Nancy L. Keating, Jordan Everson

**Affiliations:** 1 Department of Health Policy, Vanderbilt University School of Medicine, Nashville, Tennessee; 2 Department of Pharmacotherapy, University of Utah College of Pharmacy, Salt Lake City; 3 Department of Health Policy and Management, Harvard T.H. Chan School of Public Health, Boston, Massachusetts; 4 Department of Health Care Policy, Harvard Medical School, Boston, Massachusetts; 5 Division of General Internal Medicine, Brigham and Women’s Hospital, Boston, Massachusetts; 6 Office of Technology, Office of the National Coordinator for Health Information Technology, Washington, DC

## Abstract

**Importance:**

Rising prescription drug costs and increasing prices for consumer goods may increase cost-related medication nonadherence. Cost-conscious prescribing can be supported by real-time benefit tools, but patient views on real-time benefit tool use and their potential benefits and harms are largely unexplored.

**Objective:**

To assess older adults’ cost-related medication nonadherence, cost-coping strategies, and views on the use of real-time benefit tools in clinical practice.

**Design, Setting, and Participants:**

A weighted, nationally representative survey of adults aged 65 years and older administered via the internet and telephone from June 2022 to September 2022.

**Main Outcomes and Measures:**

Cost-related medication nonadherence; cost coping strategies; desire for cost conversations; potential benefits and harms from real-time benefit tool use.

**Results:**

Among 2005 respondents, most were female (54.7%) and partnered (59.7%); 40.4% were 75 years or older. Cost-related medication nonadherence was reported by 20.2% of participants. Some respondents used extreme forms of cost-coping, including foregoing basic needs (8.5%) or going into debt (4.8%) to afford medications. Of respondents, 89.0% reported being comfortable or neutral about being screened before a physician's visit for wanting to have medication cost conversations and 89.5% indicated a desire for their physician to use a real-time benefit tool. Respondents expressed concern if prices were inaccurate, with 49.9% of those with cost-related nonadherence and 39.3% of those without reporting they would be extremely upset if their actual medication price was more than what their physician estimated with a real-time benefit tool. If the actual price was much more than the estimated real-time benefit tool price, nearly 80% of respondents with cost-related nonadherence reported that it would affect their decision to start or keep taking a medication. Furthermore, 54.2% of those with any cost-related nonadherence and 30% of those without reported they would be moderately or extremely upset if their physicians used a medication price tool but chose not to discuss prices with them.

**Conclusions and Relevance:**

In 2022, approximately 1 in 5 older adults reported cost-related nonadherence. Real-time benefit tools may support medication cost conversations and cost-conscious prescribing, and patients are enthusiastic about their use. However, if disclosed prices are inaccurate, there is potential for harm through loss of confidence in the physician and nonadherence to prescribed medications.

## Key Points

**Question:**

What is the prevalence of cost-related medication nonadherence among adults aged 65 years and older and what are patients’ views on real-time prescription benefit tools?

**Findings:**

In a national panel survey of 2005 respondents in 2022, 20.2% reported any cost-related medication nonadherence. In addition, 89.5% expressed interest in their physician using real-time prescription benefit tools, although some indicated concern about their physician using a tool without discussing the prices shown and that inaccurate estimates could lead to medication nonadherence.

**Meaning:**

These findings suggest prescription drug affordability is a pressing issue.

## Teaser

This survey study evaluates cost-related medication nonadherence and desire for medication cost information among adults aged 65 years and older in the US in 2022.

## Introduction

Cost-related medication nonadherence is prevalent in the US. In a 2022 national opinion poll, 18% of adults of all ages reported not filling a prescription medication due to costs in the past year.^1^ Even among older adults insured under Medicare, 14.4% reported cost-related medication nonadherence in 2016.^2^ Recent high levels of inflation may have worsened cost-related medication nonadherence among older adults, even among those with insurance.

Although prescription drug affordability is a challenge, there are important opportunities for clinical intervention to help patients avoid cost-related nonadherence.^3^ This includes clinicians engaging in cost-conscious prescribing by identifying opportunities for improving medication affordability, and discussing costs with patients to avoid sticker shock when filling medications at the pharmacy.^4,5,6^ Real-time benefit tools that present patient-specific drug price information (ie, the patient’s expected out-of-pocket costs) to prescribers at the point of prescribing are one option that may facilitate both choosing lower-priced drugs^7,8^ and engaging in informed cost conversations.

Real-time benefit tools have been required for Medicare Part D since 2021, and one large vendor has described widespread availability of the tools.^9^ Unlike other policy interventions aimed at lowering drug costs,^1^ patient views on whether and how real-time benefit tools should be used to inform prescribing and the potential benefits and harms of their use are largely unexplored. Using a national panel, we surveyed adults aged 65 years and older to understand current cost-related medication nonadherence, medication cost coping strategies, and patients’ views on the use of real-time benefit tools to address medication costs and help inform medication cost conversations.

## Methods

### Survey Methodology

This survey study was reviewed and approved by the Vanderbilt University Medical Center institutional review board and reporting followed the American Association for Public Opinion Research (AAPOR) reporting guideline. The survey was fielded among US adults aged 65 years and older by survey firm SSRS via their Opinion Panel, a nationally representative probability-based panel of US adults aged 18 years and older, recruited via the US Postal Service’s Computerized Delivery Sequence File.^10^

Survey data for this study were collected in English and Spanish via the web or via telephone from July 14, 2022, to September 10, 2022, resulting in 4158 panel members invited to participate, with a response rate (RR3)^11^ of 48.5%. Agreement to participate in the survey conveyed consent. Participants with less than a high school education, who completed the survey by phone or in Spanish were compensated $10 while all other participants were compensated $5 for survey completion.

The collected data were weighted to represent the residential US population aged 65 years and older using a base weight and weighting benchmarks for selected demographic categories including sex by age, education, race and ethnicity and US Census region, civic engagement, population density, political party, voter registration, religious affiliation, and frequency of internet use. eTable 1 in Supplement 1 compares the sample with the benchmark parameters and the final weighted database; eTable 2 in Supplement 1 includes a description of the demographic benchmarks used in the weighting process.

### Measures

#### Cost-Related Nonadherence and Cost-Coping Strategies

The study’s primary outcome was cost-related medication nonadherence,^2,12^ a composite of 5 questions indicating whether the respondent or someone in their household did any of the following to save money on their prescription medications: decided not to fill a prescription, skipped medication doses, took less medicine, delayed filling a prescription, or used someone else’s medication. We also measured medication cost-coping activities reported by respondents including using copayment cards (eg, GoodRx), asking the physician for a lower-cost medication or free samples, shopping around at pharmacies for a lower price, attempting to find financial assistance, and purchasing prescriptions from another country. Finally, we assessed more profound cost-coping strategies including spending less money on food, heat, or other basic needs to have money for medicine and borrowing money or going into debt to help with prescription medication costs.

#### Desire for Cost Conversations and Real-time Benefit Tool Use

We developed several measures to assess respondent comfort with cost conversations and the use of real-time benefit tools. First, we asked respondents to report their comfort level for being screened before their physician’s visit regarding having a medication cost conversation. We also asked about their experience with prior cost conversations and their desire for their physicians to use real-time benefit tools and discuss medication prices with them during a visit.

#### Implementation Concerns for Real-time Benefit Tool Use

To assess potential challenges with real-time benefit tool use, we evaluated how respondents would feel if the physician used the tool but did not discuss the price information with them, and if the actual medication price at the pharmacy was higher than their physician estimated when using the tool. For the latter question, we asked whether a large price difference would affect their decision to start or keep taking their medications, their opinion of the physician using real-time benefit tools in the future, or their level of confidence in their physician. Finally, we assessed cost-coping strategies that respondents would use if the medication price was too high at the pharmacy. A complete version of the survey is available in the eAppendix in Supplement 1.

### Statistical Analysis

We described cost-related nonadherence and cost-coping strategies and compared baseline characteristics (categorized in Table 1) for respondents reporting any vs no cost-related nonadherence using weighted χ^2^ tests. We described the desire for medication cost conversations and real-time benefit tool use, concerns regarding real-time benefit tool use, and cost-coping strategies respondents expected to use if the medication price was too high at the pharmacy overall and separately for those with and without cost-related nonadherence using weighted χ^2^ tests. Two-sided *P* values of <.05 were considered statistically significant. All analyses used population weights to represent the US population aged 65 years or older. Analyses were completed using SAS Studio release 3.8 (SAS Institute).

**Table 1.** Baseline Characteristics Overall and by Reports of Cost-Related Nonadherence

Demographics	Participants, No (weighted %)^a^			*P* value
	All (N = 2005)	Any Cost-Related Nonadherence (n = 379)	No Cost-Related Nonadherence (n = 1626)	
Age, y				
65-69	644 (31.1)	153 (41.4)	491 (28.4)	<.001
70-74	608 (28.6)	119 (30.0)	489 (28.2)	
≥75	752 (40.4)	107 (28.6)	645 (43.3)	
Reported sex				
Male	942 (45.1)	154 (42.1)	788 (45.8)	.57
Female	1060 (54.7)	224 (57.6)	836 (53.9)	
Education				
Less than high school	40 (7.6)	9 (9.4)	31 (7.2)	<.001
High school graduate	374 (35.7)	71 (35.3)	303 (35.7)	
Some college	579 (22.0)	147 (29.6)	432 (20.1)	
College graduate	1011 (34.6)	151 (25.1)	860 (37.0)	
Relationship status				
Partnered	1142 (59.7)	226 (63.2)	916 (58.9)	.12
Not partnered	863 (40.3)	153 (36.9)	710 (41.1)	
Census region				
Northeast	376 (18.7)	73 (18.2)	303 (18.8)	.36
North central	437 (21.6)	88 (22.3)	349 (21.4)	
South	741 (37.9)	135 (40.6)	606 (37.2)	
West	451 (21.9)	83 (19.0)	368 (22.7)	
Income per y, $				
<15 000	98 (9.0)	19 (9.0)	79 (9.0)	<.001
15 000 to 24 999	186 (10.9)	47 (14.4)	139 (10.0)	
25 000 to 49 999	527 (29.6)	136 (38.8)	391 (27.2)	
50 000 to 99 999	752 (34.3)	130 (28.4)	622 (35.8)	
≥100 000	427 (15.4)	45 (8.8)	382 (17.1)	
Unspecified or unreported	15 (0.8)	2 (0.6)	13 (0.8)	
Household finances				
Not enough or just enough to meet basic expenses	265 (17.2)	116 (34.8)	149 (12.9)	<.001
Meets expenses with a little left over	723 (37.6)	178 (46.2)	545 (35.5)	
Lives comfortably	1013 (45.2)	83 (19.0)	930 (51.7)	
Confidence in ability to pay medical costs				
Not confident	98 (7.3)	52 (18.7)	46 (4.4)	<.001
Somewhat confident	523 (28.0)	190 (47.8)	333 (23.0)	
Very confident	1380 (64.7)	137 (33.5)	1243 (72.6)	
Need assistance with written health information				
Always or sometimes needs help	138 (10.6)	51 (18.9)	87 (8.6)	<.001
Rarely needs help	354 (18.5)	80 (21.0)	274 (17.9)	
Never needs help	1513 (70.8)	248 (60.1)	1265 (73.5)	
Health related measures				
Self-reported health				
Excellent, very good, good	1691 (79.1)	277 (67.4)	1414 (82.0)	<.001
Fair, poor	314 (20.9)	102 (32.6)	212 (18.0)	
Monthly medications used				
None	152 (8.5)	16 (5.5)	136 (9.2)	<.001
1	195 (9.5)	24 (6.7)	171 (10.2)	
2	275 (12.9)	36 (8.7)	239 (14.0)	
3	294 (14.8)	51 (13.2)	243 (15.2)	
4	251 (12.6)	54 (15.6)	197 (11.9)	
5	239 (11.6)	46 (8.9)	193 (12.2)	
≥6	593 (30.0)	151 (41.4)	442 (27.2)	
Spending on medications per mo, $				
<25	833 (42.4)	78 (20.4)	755 (47.9)	<.001
25 to <50	499 (25.7)	103 (28.8)	396 (24.9)	
50 to <100	356 (16.9)	87 (20.6)	269 (15.9)	
≥100	316 (15.0)	111 (30.1)	205 (11.2)	
Chronic conditions, No.^b^				
None	108 (5.2)	8 (1.8)	100 (6.0)	<.001
1	218 (12.2)	22 (6.3)	196 (13.7)	
2	329 (16.0)	34 (9.6)	295 (17.6)	
3	399 (20.0)	72 (19.7)	327 (20.1)	
4	377 (17.5)	72 (17.0)	305 (17.6)	
5	258 (12.4)	65 (14.8)	193 (11.7)	
≥6	316 (16.8)	106 (30.8)	210 (13.3)	

^a^ Sample weighted to represent US population aged 65 years and older.

^b^ See eTable 3 in Supplement 1 for conditions included.

## Results

Among the 2005 survey respondents, 20.2% reported cost-related medication nonadherence (because percentages are weighted, raw numbers have not been reported along with them; see Table 1 for corresponding numbers of respondents). Most respondents were female (54.7%) and partnered (59.7%). The largest portions lived in the US South Census region (37.9%) and were 75 years or older (40.4%) (Table 1). Those reporting any cost-related nonadherence were younger (aged 65-69; 41.4% vs 28.4%), less likely to be college graduates (25.1% vs 37%), and more likely to report lower incomes compared with those reporting no cost-related nonadherence.

Respondents with any cost-related nonadherence were nearly 3 times as likely to report not having enough income or having just enough income to meet basic expenses (34.8% vs 12.9%), over 4 times as likely to report not being confident they could pay their medical costs (18.7% vs 4.4%), and over twice as likely to report always or sometimes needing help with written health information (18.9% vs 8.6%) compared with those with no reported cost-related nonadherence. Respondents with cost-related nonadherence were more likely to report fair or poor health (32.6% vs 18.0%), taking 6 or more medications per month (41.4% vs 27.2%), and having 6 or more chronic conditions (30.8% vs 13.3%) (see eTable 3 in Supplement 1 for a list of conditions).

### Cost-Related Nonadherence and Cost-Coping Strategies

**Figure 1.**
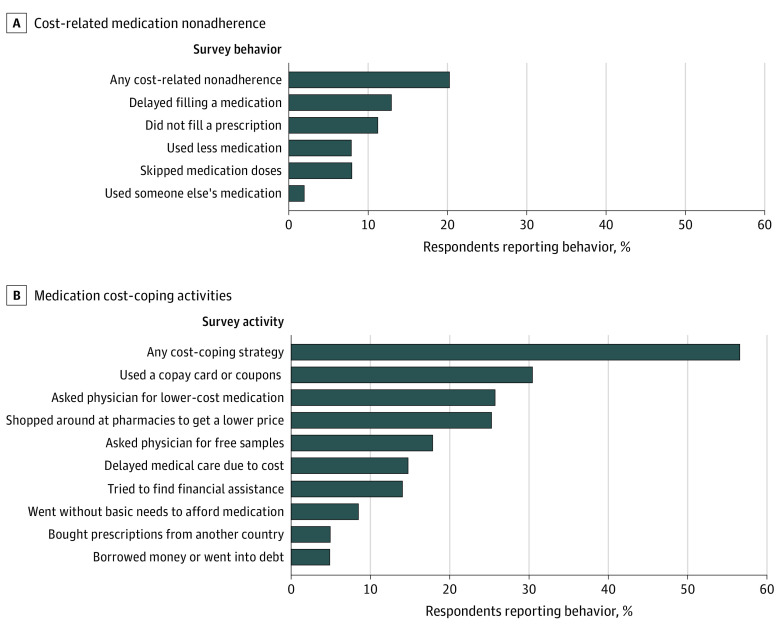
Reports of Cost-Related Medication Nonadherence and Cost Coping Activities in 2022 Among Adults Aged 65 Years and Older Sample weighted to represent US population aged 65 years and older. Respondents asked to indicate any of the described activities taken to save money on their prescription medications.

Commonly endorsed forms of cost-related nonadherence included delaying prescription fills (12.9%), not filling a prescription (11.1%), taking less medication or skipping doses (7.9% each), and using someone else’s medication (1.9%), with many respondents reporting more than one form of cost-related nonadherence (Figure 1A).

Just over half of all respondents (56.5%) used medication cost-coping strategies (Figure 1B). Cost-coping strategies included using a copayment card or coupon (30.4%), asking a physician for lower-cost medications (25.6%), shopping around at pharmacies to get a lower price (25.2%), asking a physician for free samples (17.8%), and buying prescriptions from another country (4.8%). Some respondents reported going without basic needs to afford medication (8.5%) or borrowing money or going into debt (4.8%).

### Desire for Cost Conversations and Real-time Benefit Tool Use

In examining respondents’ desire for cost conversations and the use of real-time benefit tools in clinical encounters (Table 2), 89.0% reported being comfortable or neutral (neither uncomfortable nor comfortable) about being screened before a physician’s visit regarding their interest in engaging in a medication cost conversation. Those reporting any cost-related nonadherence were more likely to report being very or somewhat uncomfortable with screening, relative to those with no cost-related nonadherence (15.3% vs 9.6%). When asked if they would like to speak to their physician about the price of their medications, 40.3% of respondents said yes, 39.0% said it would depend on the medication, 10.5% said no, and 10.2% said they were unsure. Those reporting cost-related nonadherence were more likely to respond “yes” regarding their desire to speak to their physician about the price of their medications than those without cost-related nonadherence (55.3% vs 36.5%). Additionally, those with any cost-related nonadherence were nearly twice as likely to report having had a prior cost conversation with their physician compared with those reporting no cost-related nonadherence (80.6% vs 41.0%). Overall, 89.5% of respondents indicated a desire for physicians to use real-time benefit tools; 89.8% also indicated a desire to discuss the estimated price(s), with greater interest among those with any cost-related nonadherence vs none (94.8% vs 88.5%).

**Table 2.** Desire for Medication Cost Conversations and Real-time Benefit Tool Use Overall and by Cost-Related Nonadherence Status

Desire	No. (weighted %)^a^			*P* value
	All	Any cost-related nonadherence	No cost-related nonadherence	
Comfort with cost conversation screening				
Very uncomfortable	80 (4.1)	18 (4.4)	62 (4.0)	<.001
Somewhat uncomfortable	120 (6.7)	39 (10.9)	81 (5.6)	
Neither uncomfortable nor comfortable	503 (24.0)	69 (17.0)	434 (25.7)	
Somewhat comfortable	333 (18.1)	77 (24.7)	256 (16.5)	
Very comfortable	966 (46.9)	175 (42.9)	791 (47.9)	
Desire to talk with physician about the price of medications				
Yes	822 (40.3)	220 (55.3)	602 (36.5)	<.001
It depends on the medication	858 (39.0)	128 (34.7)	730 (40.1)	
No	163 (10.5)	10 (4.2)	153 (12.0)	
Not sure	160 (10.2)	21 (5.8)	139 (11.3)	
Prior cost conversation				
Yes	1020 (49.0)	310 (80.6)	710 (41.0)	<.001
No	985 (51.0)	69 (19.4)	916 (59.0)	
Desire for physician to use real-time benefit tools^b^				
Yes	1832 (89.5)	357 (94.9)	1475 (88.2)	<.001
No	169 (10.1)	20 (4.5)	149 (11.5)	
Desire for physician to discuss real-time benefit tool price				
Yes	1832 (89.8)	364 (94.8)	1468 (88.5)	.002
No	173 (10.2)	15 (5.3)	158 (11.5)	

^a^ Sample weighted to represent US population aged 65 years and older.

^b^ Because people may be unfamiliar with the concept of real-time benefit tools, the following prompt was included to explain the meaning of this term: “Imagine your doctor could use a tool in your electronic medical record during your visit that showed them an estimate of the price that you would pay for a medication, and medication alternatives, based on your insurance.”

### Implementation Concerns for Real-time Benefit Tool Use

When considering the implementation of real-time benefit tools in clinical encounters, respondents shared a range of concerns about how they might feel if a physician used a real-time benefit tool to estimate their medication’s price but did not discuss the price, with 15.5% reporting they would be extremely upset and 20.7% not at all upset (Table 3). Those with cost-related nonadherence were more likely to report being “extremely upset” if the physician used a real-time benefit tool to estimate a medication’s price but did not discuss the price with them (23.7%) compared with those reporting no cost-related nonadherence (13.4%). Overall, 54.2% of those with any cost-related nonadherence and 30% of those without reported they would be moderately or extremely upset if their physicians used a medication price tool but chose not to discuss prices with them.

**Table 3.** Concerns Regarding Real-time Benefit Tool Use Overall and by Cost-Related Nonadherence Status

Concern	No. (weighted %)^a^			*P* value
	All	Any cost-related nonadherence	No cost-related nonadherence	
Feelings if physician used a real-time benefit tool to estimate price but did not discuss the price^b^				
Not at all upset	356 (20.7)	34 (10.2)	322 (23.3)	<.001
Slightly upset	365 (17.3)	60 (12.0)	305 (18.6)	
Somewhat upset	545 (27.1)	97 (23.4)	448 (28.1)	
Moderately upset	434 (19.4)	102 (30.5)	332 (16.6)	
Extremely upset	304 (15.5)	85 (23.7)	219 (13.4)	
Feelings if price was a lot more than the physician estimated with a real-time benefit tool				
Not at all upset	68 (4.2)	8 (1.5)	60 (4.9)	<.001
Slightly upset	155 (7.6)	21 (5.7)	134 (8.1)	
Somewhat upset	448 (20.9)	65 (15.9)	383 (22.2)	
Moderately upset	550 (25.4)	101 (26.9)	449 (25.1)	
Extremely upset	777 (41.5)	182 (49.9)	5695 (39.3)	
Affect decision to start or keep taking medication if price was a lot more than estimated				
Yes	986 (53.7)	289 (78.1)	697 (47.5)	<.001
No	1015 (46.1)	90 (21.9)	925 (52.2)	
Opinion of physician using the real-time benefit tool if price was a lot more than estimated				
Still want my physician to use the tool	916 (42.4)	187 (49.3)	729 (40.7)	.002
Only use the tool if they thought the price might be very high	597 (30.1)	89 (21.4)	508 (32.3)	
Would not want my physician to use the tool again	488 (27.0)	103 (29.3)	385 (26.5)	
Confidence in physician if price was a lot more than estimated				
Decrease my confidence in my physician a lot	184 (10.4)	44 (15.1)	140 (9.2)	.003
Decrease my confidence in my physician a little	687 (33.3)	130 (35.5)	557 (32.8)	
Would not decrease my confidence in my physician	1130 (56.1)	204 (49.3)	926 (57.9)	
If the real-time benefit tool showed average price vs specific price, would you want that information				
Yes	1676 (82.6)	323 (85.9)	1353 (81.8)	.04
No	325 (17.1)	55 (13.3)	270 (18.0)	

^a^ Sample weighted to represent US population aged 65 years and older.

^b^ Because people may be unfamiliar with the concept of real-time benefit tools, the following prompt was included to explain the meaning of this term: “Imagine your doctor could use a tool in your electronic medical record during your visit that showed them an estimate of the price that you would pay for a medication, and medication alternatives, based on your insurance.”

Across all respondents, 41.5% reported they would be extremely upset if there were large discrepancies between prices provided by the real-time benefit tool and the actual price paid at the pharmacy. Those with cost-related nonadherence were more likely to report being “extremely upset” if the price was more than what their physician estimated with a real-time benefit tool (49.9%) compared with those reporting no cost-related nonadherence (39.3%). Furthermore, if the actual price was much more than the estimated real-time benefit tool price, 78.1% of respondents with cost-related nonadherence reported that it would affect their decision to start or keep taking a medication (vs 47.5% of respondents with no cost-related nonadherence). Additionally, 43.7% of respondents stated that their confidence in their physician would decrease if the actual price was much more than the real-time benefit tool estimated price that was discussed, with similar responses for those with (50.6%) and without (42.0%) any cost-related nonadherence. Finally, 82.6% of respondents noted they would be interested in seeing the average price of the drug if their specific price was not available.

**Figure 2.**
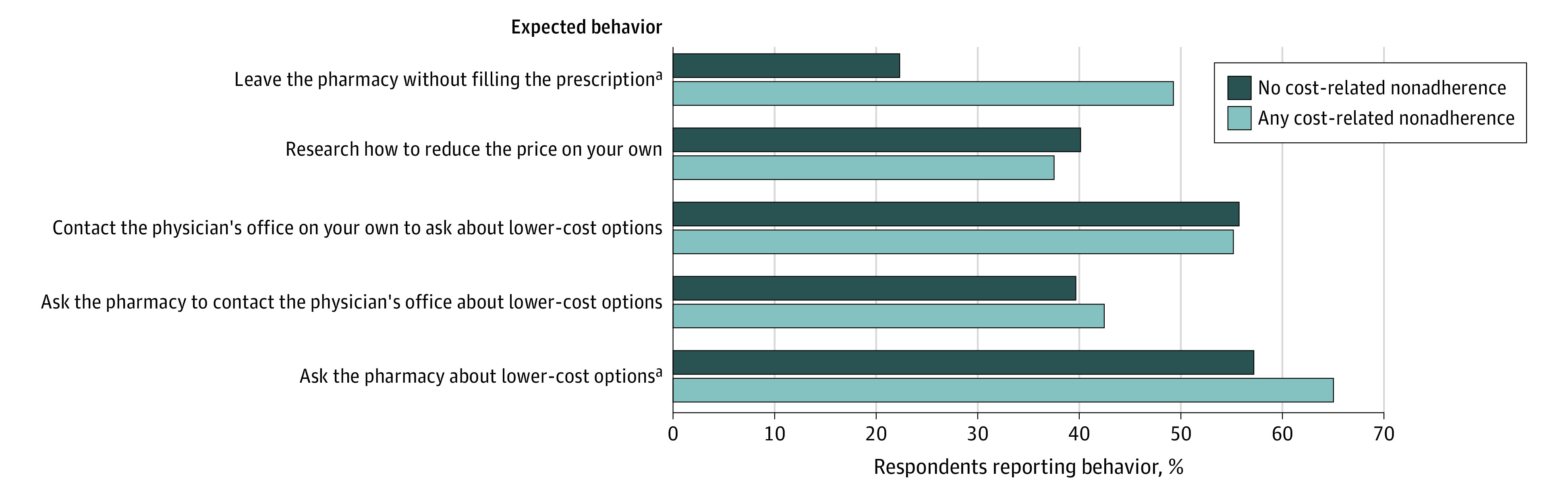
Behaviors Reported if a Medication Price was Too High at the Pharmacy, Overall and by Cost-Related Nonadherence in 2022 Among Adults Aged 65 Years and Older Sample weighted to represent US population aged 65 years and older. ^a^*P* < .05.

Lastly, respondents reported about their expected behavior if they arrived at the pharmacy and their medication cost was too high (Figure 2). Respondents with cost-related nonadherence were more likely to report that they would leave the pharmacy without filling a prescription compared with those reporting no cost-related nonadherence (49.2% vs 22.2%). Otherwise, responses were similar across those with and without cost-related nonadherence.

## Discussion

Among a nationally representative sample of individuals aged 65 years and older, approximately 1 in 5 reported experiencing cost-related medication nonadherence in the past year. This is similar to other recently published reports^1^ and somewhat higher than surveys of Medicare beneficiaries from prior years.^2^ The high levels of general inflation, which rose from 1.4% in January 2021 to 9.1% in June 2022,^13^ may be contributing to greater reports of cost-related nonadherence than in prior years. Importantly, cost coping strategies may be preventing some forms of cost-related nonadherence but are themselves concerning, with 8.5% of respondents in our survey reporting going without basic needs to afford medications.

There has been growing interest in addressing cost-related nonadherence through price-conscious prescribing and engaging in meaningful cost conversations at the point of prescribing.^6,14,15^ By providing patient-specific drug prices to prescribers, real-time benefit tools could ensure that prescribers are aware of the price of drugs and their alternatives, thereby addressing a key barrier to both price-conscious prescribing and cost conversations.^7,16^ We found that 89.5% of respondents would want their physician to use a real-time benefit tool if it was available to them and that most of those who want their physician to use a real-time benefit tool want their physician to talk with them about their estimated prices for medications. Respondents described varied levels of distress if their physician used the real-time benefit tool but did not discuss prices with them, with about equal proportions saying they would not be upset as said they would be very upset. These findings highlight a risk that real-time benefit tools, if used by clinicians but not discussed with patients, could drive prescribing decisions that are not patient-centered but rather reflect prescribers’ assumptions about their patient’s needs.^17^

We found that nearly all (89.0%) respondents said they would be comfortable or neutral regarding being screened for their interest in a medication cost conversation before their physician’s visit. Prior studies have shown that physicians infrequently initiate cost conversations^18,19,20,21^ and are concerned that patients may be uncomfortable with discussing treatment costs.^6,21^ Asking patients about their interest in this topic on their clinic intake form may be one way to know if this is a topic of interest for the patient for the current visit.^22^

Unfortunately, the implementation of real-time benefit tools is challenged by potentially incomplete data (eg, interoperability challenges between prescription drug plan data from insurers and electronic health records),^23,24^ and the accuracy of these tools is unclear. Real-time benefit tool estimates are meant to be specific to a patient, pharmacy, and time, and if a patient waits to fill a prescription or chooses to go to a different pharmacy, their medication price can be different. We asked individuals about their views in situations in which the price provided via the real-time benefit tool was different from the price quoted at the pharmacy and found that patients reported that this discrepancy would be very upsetting, would reduce their confidence in their physician, and would reduce their likelihood of filling their medication. This finding indicates the potential that the use of a technology such as a real-time benefit tool to estimate drug prices could create a misleading impression of precise prices and negatively impact the physician-patient relationship. Despite this, respondents were eager to have their physicians use real-time benefit tools and to discuss the resulting prices with them.

Given the potential pitfalls to real-time benefit tool use, what might physicians do to best meet patient needs while acknowledging the potential inaccuracies in estimates available today? First, our findings show widespread support for the use of real-time benefit tools, such that health systems, developers of health information technology, and policy makers should follow through on initiatives to make these tools available to all prescribers. One recent study demonstrated that even when available, such tools are rarely used without focused marketing or training efforts.^25^ Our results suggest that health system leaders should invest in those activities.

Second, physicians should use these tools when available within the context of cost conversations. Although the complexity of prescribing decisions can vary from simple formulation changes to decisions with more direct trade-offs, our findings should encourage prescribers to default to conversations whenever possible.^26^ Importantly, physicians may wish to indicate to patients that the prices given are estimates and may be different at the pharmacy, and to discuss what patients should do if the price discrepancy is large. For example, telling patients to have the pharmacist contact the physician if the price is too high would be one way to highlight and prepare the patient for the potential for a price discrepancy and what to do if this occurs. One challenge to these conversations is that while patients are supportive of the use of real-time benefit tools and want to discuss costs, their specific concerns and sensitivity to price differs substantially, so that effective conversations must account for these differences.^26,27^ Additional research is warranted to better understand how patients and physicians would like to engage with these tools in practice and how best to communicate price estimates in a way that minimize harms and maximize benefits.

Third, physicians should consider engaging other health care team members (eg, pharmacists, nurses) to support medication cost discovery and cost conversations. Most respondents already considered asking the pharmacy for lower-cost options in a scenario in which they discover that a medication price is too high for them. Use of real-time benefit tools and consultation with pharmacists in advance of the patient’s arrival at the pharmacy could prevent inefficiencies that result from patients needing medication changes once they are at the pharmacy counter. This proactive approach also reduces the burden on patients and caregivers, reduces trips to the pharmacy, and could ultimately prevent cost-related nonadherence.^28,29^

### Limitations

This analysis has limitations. First, our survey is subject to nonresponse bias. Nevertheless, we surveyed individuals who were part of a nationally representative panel survey, achieved a response rate of nearly 50%, and weighted our data to reflect the US population aged 65 years and older. Moreover, our estimates of cost-related nonadherence are similar to other recent estimates. Second, our findings may not generalize to younger individuals who may experience cost-related nonadherence, especially if health conditions interfere with employment and employer-sponsored insurance. We also cannot be certain that individuals’ responses reflect actual behaviors and beliefs. Third, we were broadly interested in the experience of cost-related nonadherence and were unable to address the types (eg, chronic, acute, specialty), frequency, costs, or health consequences associated with foregone medications. These are important areas for future research. Fourth, real-time benefit tools may increase cost-conscious prescribing and better prepare patients for the prices they will face at the pharmacy counter, but they do not reduce drug prices directly. Broader efforts and policy reforms are needed to lower the prices of medications and to improve insurance coverage.

## Conclusion

In 2022, approximately 1 in 5 older adults reported cost-related nonadherence. Real-time benefit tools may support medication cost conversations and cost-conscious prescribing, and patients are enthusiastic about their use. However, if disclosed prices are inaccurate there is potential for harm through loss of confidence in the physician and nonadherence to prescribed medications.

## Notes

Supplement 1. **eTable 1. **Demographic Benchmarks, Sample Characteristics, and Weighted Demographics **eTable 2. **Sample Disposition and Response Rates **eTable 3. **Medical Conditions **eAppendix. **Medication Cost Conversations and Perceptions of a Real Time Benefit Tool National Patient Survey

Supplement 2.
Data Sharing Statement

## References

[1] Hamel L, Lopes L, Kirzinger A, . Public opinion on prescription drugs and their prices. KFF. 2022. Accessed November 29, 2022. https://www.kff.org/health-costs/poll-finding/public-opinion-on-prescription-drugs-and-their-prices/

[2] Nekui F, Galbraith AA, Briesacher BA, . Cost-related medication nonadherence and its risk factors among medicare beneficiaries. Med Care. 2021;59(1):13-21. doi:10.1097/MLR.0000000000001458 33298705 PMC7735208

[3] Patel MR, Jagsi R, Resnicow K, . A scoping review of behavioral interventions addressing medical financial hardship. Popul Health Manag. 2021;24(6):710-721. doi:10.1089/pop.2021.0043 33989065 PMC8713277

[4] Harrington NG, Scott AM, Spencer EA. Working toward evidence-based guidelines for cost-of-care conversations between patients and physicians: a systematic review of the literature. Soc Sci Med. 2020;258:113084. doi:10.1016/j.socscimed.2020.113084 32569948

[5] Hamel LM, Dougherty DW, Hastert TA, . The DISCO app: a pilot test of a multi-level intervention to reduce the financial burden of cancer through improved cost communication. PEC Innov. 2022;1:100002. doi:10.1016/j.pecinn.2021.100002 PMC10194252 37364004

[6] Everson J, Henderson SC, Cheng A, Senft N, Whitmore C, Dusetzina SB. Demand for and occurrence of medication cost conversations: a narrative review. Med Care Res Rev. 2023;80(1):16-29. doi:10.1177/10775587221108042 35808853

[7] Desai SM, Chen AZ, Wang J, . Effects of real-time prescription benefit recommendations on patient out-of-pocket costs: a cluster randomized clinical trial. JAMA Intern Med. 2022;182(11):1129-1137. doi:10.1001/jamainternmed.2022.3946 36094537 PMC9468947

[8] Siwicki B. UCHealth changes prescribing behavior for the better with real-time transparency data. Healthcare IT News. May 19, 2022. Accessed March 25, 2023. https://www.healthcareitnews.com/news/uchealth-changes-prescribing-behavior-better-real-time-transparency-data

[9] Surescripts. 2021 National progress report. 2022. Accessed November 21, 2022. https://surescripts.com/docs/default-source/national-progress-reports/2021-national-progress-report.pdf

[10] SSRS. SSRS opinion panel. Accessed November 1, 2022. https://ssrs.com/opinion-panel/

[11] American Association for Public Opinion Research. Standard definitions. 2016. Accessed April 15, 2023. https://www.aapor.org/AAPOR_Main/media/publications/Standard-Definitions20169theditionfinal.pdf

[12] Soumerai SB, Pierre-Jacques M, Zhang F, . Cost-related medication nonadherence among elderly and disabled medicare beneficiaries: a national survey 1 year before the medicare drug benefit. Arch Intern Med. 2006;166(17):1829-1835. doi:10.1001/archinte.166.17.1829 17000938

[13] CoinNews Media Group, LLC. Current US inflation rates: 2000-2022. July 23, 2008. Accessed November 30, 2022. https://www.usinflationcalculator.com/inflation/current-inflation-rates/

[14] Donohue JM, Huskamp HA, Wilson IB, Weissman J. Whom do older adults trust most to provide information about prescription drugs? Am J Geriatr Pharmacother. 2009;7(2):105-116. doi:10.1016/j.amjopharm.2009.04.005 19447363 PMC2782479

[15] Fiscella K, Venci J, Sanders M, Lanigan A, Fortuna R. A practical approach to reducing patients’ prescription costs. Fam Pract Manag. 2019;26(3):5-9. 31083870

[16] Mummadi SR, Mishra R. Effectiveness of provider price display in computerized physician order entry (CPOE) on healthcare quality: a systematic review. J Am Med Inform Assoc. 2018;25(9):1228-1239. doi:10.1093/jamia/ocy076 29982523 PMC7646898

[17] Dullabh P, Heaney-Huls K, Lobach DF, . The technical landscape for patient-centered CDS: progress, gaps, and challenges. J Am Med Inform Assoc. 2022;29(6):1101-1105. doi:10.1093/jamia/ocac029 35263437 PMC9093031

[18] Hunter WG, Zafar SY, Hesson A, . Discussing health care expenses in the oncology clinic: analysis of cost conversations in outpatient encounters. J Oncol Pract. 2017;13(11):e944-e956. doi:10.1200/JOP.2017.022855 28834684 PMC5684881

[19] Ubel PA, Zhang CJ, Hesson A, . Study of physician and patient communication identifies missed opportunities to help reduce patients’ out-of-pocket spending. Health Aff (Millwood). 2016;35(4):654-661. doi:10.1377/hlthaff.2015.1280 27044966 PMC5960072

[20] Tarn DM, Paterniti DA, Heritage J, Hays RD, Kravitz RL, Wenger NS. Physician communication about the cost and acquisition of newly prescribed medications. Am J Manag Care. 2006;12(11):657-664. 17090222

[21] Schrag D, Hanger M. Medical oncologists’ views on communicating with patients about chemotherapy costs: a pilot survey. J Clin Oncol. 2007;25(2):233-237. doi:10.1200/JCO.2006.09.2437 17210946

[22] Sloan CE, Gutterman S, Davis JK, . How can healthcare organizations improve cost-of-care conversations? A qualitative exploration of clinicians’ perspectives. Patient Educ Couns. 2022;105(8):2708-2714. doi:10.1016/j.pec.2022.04.005 35440376

[23] Everson J, Dusetzina SB. Real-time prescription benefit tools-the promise and peril. JAMA Intern Med. 2022;182(11):1137-1138. doi:10.1001/jamainternmed.2022.3962 36094566

[24] Everson J, Frisse ME, Dusetzina SB. Real-time benefit tools for drug prices. JAMA. 2019;322(24):2383-2384. doi:10.1001/jama.2019.16434 31651954

[25] Bhardwaj S, Miller SD, Bertram A, Smith K, Merrey J, Davison A. Implementation and cost validation of a real-time benefit tool. Am J Manag Care. 2022;28(10):e363-e369. doi:10.37765/ajmc.2022.89254 36252176

[26] Everson J, Whitmore CC, Mattingly TJ II, Sinaiko AD, Dusetzina SB. Physician perspectives on implementation of real-time benefit tools: a qualitative study. Appl Clin Inform. 2022;13(5):1070-1078. doi:10.1055/a-1947-2674 36122592 PMC9646401

[27] Mattingly TJ II, Everson J, Besaw RJ, Whitmore CC, Henderson SC, Dusetzina SB. “Worth it if you could afford it”: patient perspectives on integrating real-time benefit tools into drug cost conversations. J Am Geriatr Soc. 2023. doi:10.1111/jgs.18226 36637794

[28] Kassamali B, Faletsky A, Han JJ, . Physician perspectives on the effect of topical steroid costs on patients and proposed solutions. JAMA Dermatol. 2022;158(1):79-83. doi:10.1001/jamadermatol.2021.4140 34668921 PMC8529518

[29] Shrank WH, Hoang T, Ettner SL, . The implications of choice: prescribing generic or preferred pharmaceuticals improves medication adherence for chronic conditions. Arch Intern Med. 2006;166(3):332-337. doi:10.1001/archinte.166.3.332 16476874

