# Supplementary material for: Cost-Related Medication Nonadherence and Desire for Medication Cost Information Among Adults Aged 65 Years and Older in the US in 2022
Source: JAMA Netw Open. 2023 May 18;6(5):e2314211. doi: 10.1001/jamanetworkopen.2023.14211 (PMC10196872; doi:10.1001/jamanetworkopen.2023.14211)
Supplement: Supplement 1. — eTable 1. Demographic Benchmarks, Sample Characteristics, and Weighted Demographics eTable 2. Sample Disposition and Response Rates eTable 3. Medical Conditions eAppendix. Medication Cost Conversations and Perceptions of a Real Time Benefit Tool National Patient Survey [file jamanetwopen-e2314211-s001.pdf]

Supplemental Online Content

Dusetzina SB, Besaw RJ, Whitmore CC, et al. Cost-related medication nonadherence and desire for medication cost information among adults aged 65 years and older in the US in 2022. *JAMA Netw Open*. 2023;6(5):e2314211. doi:10.1001/jamanetworkopen.2023.14211

eTable 1. Demographic Benchmarks, Sample Characteristics, and Weighted Demographics
eTable 2. Sample Disposition and Response Rates
eTable 3. Medical Conditions
eAppendix. Medication Cost Conversations and Perceptions of a Real Time Benefit Tool National Patient Survey

This supplemental material has been provided by the authors to give readers additional information about their work.

© 2023 Dusetzina SB et al. *JAMA Network Open.*

eTable 1: Demographic Benchmarks, Sample Characteristics, and Weighted Demographics[1]

Category	Values	Parameter	Unweighted	Weighted
Sex by age	M 65-74	27.5%	27.9%	27.1%
	M 75+	17.7%	19.2%	18.2%
	F 65-74	31.7%	34.6%	32.5%
	F 75+	23.1%	18.3%	22.1%
Education	LT HS grad	10.8%	2.0%	7.6%
	HS graduate	35.3%	18.7%	35.7%
	Some Coll/Assoc Degree	21.0%	28.9%	22.2%
	College grad+	32.8%	50.4%	34.6%
Race/Ethnicity	White, non-Hispanic	75.4%	84.7%	77.9%
	Black, non-Hispanic	9.4%	6.6%	9.0%
	Hispanic	9.0%	4.5%	7.6%
	Other/Mixed, non-Hispanic	6.2%	4.2%	5.6%
Census region	Northeast	18.5%	19.0%	18.8%
	Midwest	21.2%	21.6%	21.6%
	South	37.8%	36.6%	37.3%
	West	22.5%	22.8%	22.3%
Civic engagement	Not civically engaged	63.5%	48.1%	62.0%
	Civically engaged	36.5%	51.9%	38.0%
Population density	1 Least dense	23.2%	19.1%	23.5%
	2	20.9%	20.2%	21.5%
	3	19.9%	23.1%	19.9%
	4	18.4%	20.0%	17.9%
	5 Most dense	17.6%	17.6%	17.2%
Party ID (panel)	Rep	33.2%	27.9%	33.1%
	Dem	33.1%	39.7%	32.6%
	Ind/Other	33.7%	32.5%	34.2%
Voter Registration	Registered to vote	91.6%	95.9%	92.8%
	Not registered	8.4%	4.1%	7.2%

© 2023 Dusetzina SB et al. *JAMA Network Open.*

Category	Values	Parameter	Unweighted	Weighted
Religion	Affiliated	79.8%	77.1%	79.9%
	Not Affiliated	20.2%	22.9%	20.1%
Internet Frequency	Almost constantly	14.5%	18.8%	15.2%
	Several times a day	50.3%	65.3%	52.7%
	About once a day	11.8%	7.7%	11.9%
	Several times a week	7.3%	3.2%	7.2%
	Less often	5.4%	2.0%	4.4%
	Not an internet user	10.7%	3.0%	8.6%

© 2023 Dusetzina SB et al. *JAMA Network Open.*

eTable 2. Sample Disposition and Response Rates

Completion Rates/Composite Response Rates	Total
Total Sample (Invited to participate)	4158
Screen-outs	11
Total Eligible	4147
Quality control removals	30
Incompletes	138
Quota full	0
Completions*	2005
Incidence/Eligibility rate	99.45%
Survey Completion rate (Completions/Total invited to participate)	48.22%
Survey RR3	48.46%

**Excludes screen-outs or data quality removals that completed the survey.*

Target Population Benchmarks[1]

Data were weighted to distributions of sex by age, detailed education, race/ethnicity, census region, civic engagement, population density, political party,[2] voter registration, religious affiliation, and internet use frequency. The main demographic benchmarks were obtained from the 2021 Current Population Survey (CPS).[3] The civic engagement benchmark was derived from September 2019 CPS Volunteering and Civic Life Supplement data.[2,4] Population density was derived from Census Planning Database 2020.[5] The registered voter benchmark is from Aristotle Voter Data 2021 and Annual Estimates of the Resident Population by Single Year of Age and Sex for the United States: April 1, 2020, to July 1, 2021, from the U.S. Census Bureau.[6] The party ID, internet frequency, and religious affiliation benchmarks came from NPORS annual dataset released by Pew Research.[7] Weights were trimmed at the 2nd and 98th percentiles to prevent individual interviews from having too much influence on survey-derived estimates.

© 2023 Dusetzina SB et al. *JAMA Network Open.*

eTable 3. Medical Conditions

**Demographics**	All	Any cost-related nonadherence	No cost-related nonadherence	P-Value
Number of Chronic Conditions				<0.001
None	5.2	1.8	6.0	
1	12.2	6.3	13.7	
2	16.0	9.6	17.6	
3	20.0	19.7	20.1	
4	17.5	17.0	17.6	
5	12.4	14.8	11.7	
6+	16.8	30.8	13.3	
Chronic Conditions				
Alzheimer’s Disease / Dementia	1.5	2.0	1.4	0.39
Anxiety	20.5	30.3	18.0	<0.001
Asthma	14.9	26.5	12.0	<0.001
Cancer	25.7	22.8	26.4	0.14
Chronic Kidney Disease	7.8	10.6	6.8	0.009
Chronic Obstructive Pulmonary Disease (COPD)	11.4	20.6	9.1	<0.001
Depression	20.5	33.0	17.3	<0.001
Diabetes/Sugar Diabetes	23.1	33.4	20.5	<0.001
Glaucoma	10.2	11.2	9.9	0.47
Heart Disease / Ischemic Heart Disease	17.7	21.6	16.7	0.02
High Blood Pressure (Hypertension)	66.5	73.4	64.7	0.0009
High Cholesterol	59.8	65.2	58.4	0.0135
Irregular Heartbeat (Atrial Fibrillation)	19.0	24.9	17.5	0.0006
Osteoporosis	19.6	26.5	17.8	<0.001
Rheumatoid Arthritis / Osteoarthritis	35.9	49.5	32.4	<0.001

© 2023 Dusetzina SB et al. *JAMA Network Open.*

References for eTables.

1. Jennifer Su, Jonathan Best, Jessica Stapleton. SSRS Opinion Panel Methods Report Survey on Medication Cost Conversations and Perceptions of a Real Time Benefit Tool. Published online October 24, 2022.

2. SSRS. SSRS Full-service Survey and Market Research Firm. Accessed November 1, 2022. https://ssrs.com/

3. Ruggles S, Flood S, Goeken R, et al. IPUMS USA: Version 9.0. Published online 2019. doi:10.18128/D010.V9.0

4. US Census Bureau. Supplemental Surveys. Accessed November 1, 2022. https://www.census.gov/programs-surveys/cps/about/supplemental-surveys.html

5. US Census Bureau. 2020 Planning Database. Accessed November 1, 2022. https://www.census.gov/topics/research/guidance/planning-databases/2020.html

6. US Census Bureau. National Population by Characteristics: 2020-2021. Accessed November 1, 2022. https://www.census.gov/data/tables/time-series/demo/popest/2020s-national-detail.html

7. Pew Research Center. National Public Opinion Reference Survey (NPORS). Published online September 21, 2022. Accessed November 1, 2022. https://www.pewresearch.org/methods/fact-sheet/national-public-opinion-reference-survey-npors/

© 2023 Dusetzina SB et al. *JAMA Network Open.*

eAppendix. Medication Cost Conversations and Perceptions of a Real-Time Benefit Tool National Patient Survey

Study Overview

This survey is for a study about the cost of prescription medications and the tools that doctors and patients may use to discuss medication costs during office visits.

The study is being led by Vanderbilt University's School of Medicine and is funded by the Robert Wood Johnson Foundation, the nation's largest philanthropy dedicated solely to health.

Your participation is completely voluntary, meaning that you do not have to participate if you don't want to, and you can end your participation at any time. Your name and other identifying information will NOT be attached to the survey data given to Vanderbilt for analysis. This data will be used for research purposes only and will not be used by insurance companies, drug companies, physician offices, or any other for-profit group.

We want to thank you for taking the time to participate and look forward to learning about your experiences. Do you agree to participate in this survey?

1. **In the past 30 days, how many different prescription medications have you taken? (Fill in blank)**

2. **On average, how much do you and others in your household spend each month on your prescription medications that you fill at a pharmacy or get through mail order, including any co-pays or other out-of-pocket expenses? Do you spend:**

- ☐ Less than $25
- ☐ $25 but less than $50
- ☐ $50 but less than $100
- ☐ $100 or more a month
- ☐ Don't know / Refused

Cost-Related Barriers to Care Domain

3. **During the past 12 months, have you or someone in your household delayed medical care because you were worried about the cost?**

- ☐ Yes
- ☐ No
- ☐ Don't Know/Refused

© 2023 Dusetzina SB et al. *JAMA Network Open.*

4. During the past 12 months, was there a time when you or someone in your household needed a prescription medication but did not get it because you couldn't afford it?

☐ Yes
☐ No
☐ Don't Know/Refused

5. During the past 12 months, did you or someone in your household do any of the following things to save money on your prescription medication?

	**Yes**
Skipped medication doses to save money	
Took less medicine to save money	
Delayed filling a prescription to save money	
Used someone else's medicine	
Spent less money on food, heat, or other basic needs so that you would have money for medicine	
None of these to save money	

6. In the past 12 months, have you or someone in your household done any of the following to help with your prescription medication costs?

	**Yes**	**No**	**Don't Know**
Asked your doctor for a lower cost medication			
Bought prescription drugs from another country			
Asked your doctor for free medication samples			
Used a copay card or coupons (such as GoodRx)			
Tried to find financial assistance for your medication			
Shopped around at pharmacies to get a medication at a lower price			
Borrowed money or went into debt			

7. If you find out at the pharmacy that you could not afford the price of a medication, what would be most likely to do? Check all that apply.

☐ Go ahead and fill the prescription
☐ Ask the pharmacy about lower cost options
☐ Ask the pharmacy to contact the doctor's office to ask about lower cost options
☐ Contact the doctor's office on your own to ask about lower cost options
☐ Research how to reduce the price on your own
☐ Leave the pharmacy without filling the prescription
☐ Don't Know/Refused

© 2023 Dusetzina SB et al. *JAMA Network Open.*

Importance of Cost Conversations to Respondent Domain

8. **Have you ever discussed the price of your medications with your doctor or someone else on your healthcare team?** "Healthcare team" includes doctors, pharmacists, nurses, financial counselors, social workers, administrative workers and others.

- ☐ Yes
- ☐ No
- ☐ Don't Know

If they answered YES to "ever discussed" question

9. **The last time you discussed the price of medications with your doctor or someone else on your health care team, who did you speak with? (Choose all that apply)**

- ☐ Doctor
- ☐ Pharmacist
- ☐ Nurse/Physician Assistant
- ☐ Financial Counselor
- ☐ Social Worker
- ☐ Someone else
- ☐ Does not recall

If they answered YES to "ever discussed" question

10. **The last time you discussed the price of medications, was information about medication prices explained to you in a way that was difficult or easy to understand?**

- ☐ Very difficult to understand
- ☐ Difficult to understand
- ☐ Neither difficult nor easy to understand
- ☐ Easy to understand
- ☐ Very easy to understand
- ☐ Don't Know/Refused

If they answered YES to "ever discussed" question

11. **The last time you discussed the price of medications, did your doctor and other members of your healthcare team show respect for what you had to say about medication prices?**

- ☐ Yes, definitely
- ☐ Yes, somewhat
- ☐ No
- ☐ Don't Know/Refused

© 2023 Dusetzina SB et al. *JAMA Network Open.*

If they answered YES to "ever discussed" question

12. The last time you discussed the price of medications, did your doctor and other members of your healthcare team spend enough time discussing medication prices with you?

- ☐ Yes, definitely
- ☐ Yes, somewhat
- ☐ No
- ☐ Don't Know/Refused

13. With which healthcare professional would you feel comfortable discussing the price of your medications? (Choose all that apply)

- ☐ Doctor
- ☐ Pharmacist
- ☐ Nurse/Physician Assistant
- ☐ Financial Counselor
- ☐ Social Worker
- ☐ Someone else
- ☐ I do not want to discuss cost

14. How often would you like your doctor to consider medication price when deciding which medication to prescribe for you?

- ☐ Always
- ☐ Only when the medication price is over a certain amount.
- ☐ Never
- ☐ Don't Know/Refused

If they answered OTHER to "how often" question

What Amount?

- ☐ Less than $25 a month
- ☐ $25 but less than $50 a month
- ☐ $50 but less than $100 a month
- ☐ $100 or more a month

© 2023 Dusetzina SB et al. *JAMA Network Open.*

Potential for Real-Time Benefit Tools Domain

Imagine you went to a doctor's appointment and the following question appeared on a form you filled out when you arrived for your appointment: "Would you like to talk with your doctor about the price of your medications?" If you indicated "yes" to this question on your form, your doctor would receive an electronic reminder to review price information with you during the prescribing process.

15. How comfortable or uncomfortable would you be answering this question?

- ☐ Very uncomfortable
- ☐ Somewhat uncomfortable
- ☐ Neither uncomfortable nor comfortable
- ☐ Somewhat comfortable
- ☐ Very comfortable
- ☐ Don't Know/Refused

16. If you were asked before your visit "Would you like to talk with your doctor about the price of your medications?" how would you answer?

- ☐ "Yes, I want to talk about the price of my medications"
- ☐ "It would depend on the medication"
- ☐ "No, I do not want to talk about the price of my medications"
- ☐ I'm not sure

If they answered "It depends"

17. Which medication would you want to talk about the price of? (Check all that apply)

- ☐ Only want to talk about medications I'm already taking
- ☐ Only want to talk about new medications
- ☐ Only want to talk if lower cost alternatives exist
- ☐ Only want to talk about medications that are above a certain price
- ☐ Don't Know/Refused

Imagine your doctor could use a tool in your electronic medical record during your visit that showed them an <u>estimate</u> of the price that you would pay for a medication, and medication alternatives, based on your insurance.

18. Would you want your doctor to use that tool to <u>estimate</u> the price of your medication?

- ☐ Yes
- ☐ No
- ☐ Don't Know/Refused

© 2023 Dusetzina SB et al. *JAMA Network Open.*

19. Would you want your doctor to talk with you about the estimated medication price the tool came up with?

- ☐ Yes
- ☐ No
- ☐ Don't Know

20. If your doctor used that tool to estimate the price but did not talk about the price with you, how would you feel?

- ☐ Not at all upset
- ☐ Slightly upset
- ☐ Somewhat upset
- ☐ Moderately upset
- ☐ Extremely upset
- ☐ Don't Know/Refused

21. If you went to pick up that medication and the actual price was <u>a lot more</u> than your doctor estimated it would be when using the tool, how would you feel?

- ☐ Not at all upset
- ☐ Slightly upset
- ☐ Somewhat upset
- ☐ Moderately upset
- ☐ Extremely upset
- ☐ Don't Know/Refused

22. If the actual price of your medication was a lot more than your doctor estimated it would be, how, if at all, would that change your opinion about your doctor using the tool?

- ☐ I would still want my doctor to use the tool
- ☐ I would only want my doctor to use the tool if they thought the price might be very high
- ☐ I would not want my doctor to use the tool again
- ☐ Don't Know/Refused

23. If the actual price of your medication was a lot more than your doctor estimated it would be, how, if at all, would that affect your confidence <u>in the doctor who prescribed your medicine</u>?

- ☐ It would decrease my confidence in my doctor a lot
- ☐ It would decrease my confidence in my doctor a little
- ☐ It would not decrease my confidence in my doctor at all
- ☐ Don't Know/Refused

© 2023 Dusetzina SB et al. *JAMA Network Open.*

24. If the actual price of your medication was a lot more than your doctor estimated it would be, would it affect whether you would start or keep taking your medication?

☐ Yes
☐ No
☐ Don’t Know/Refused

25. If your doctor could not use the tool to tell you <u>your specific price</u> for a medication, would you want your doctor to use a tool that showed them <u>the average price</u> paid for a medication across all patients with health insurance? Note: Your actual price may be more or less than the average.

☐ Yes
☐ No
☐ Don’t Know/Refused

<u>Using a Patient-Facing Real-Time Benefit Tool</u>

26. If you had access to an online tool that showed you (rather than your doctor) the price you would be expected to pay for your medications, would you use it?

☐ Yes
☐ No
☐ I already use a tool that shows me the price of my medications
☐ Don’t Know

If yes or already use tool

27. When do you or would you use the tool to look at the price of medications? Check all that apply.

☐ After a conversation in which my doctor indicated that it was possible the medication would be expensive
☐ Any time I was prescribed a new medication
☐ After being prescribed any medication and before visiting the pharmacy
☐ After visiting the pharmacy and being informed of a high cost
☐ Other: ___________________
☐ Don’t Know/Refused

28. If you had the choice between EITHER your doctor looking up medication prices during a visit OR you looking up medication prices after the visit, which would you prefer?

☐ Have my doctor look up prices during the visit and make any needed changes at that time
☐ Look up prices on my own after the visit and contact my doctor to make any needed changes
☐ Don’t Know/Refused

© 2023 Dusetzina SB et al. *JAMA Network Open.*

29. If you are having trouble affording the price of a medication, which of the following would you prefer (Choose one answer):

- ☐ My doctor prescribes the option they think is best.
- ☐ My doctor gives me options, tells me their prices and how well they work, <u>and together we decide</u>.
- ☐ My doctor gives me options, tells me their prices and how well they work, <u>and lets me decide</u>.
- ☐ Other: _________________

<u>Demographics Domain</u>

30. Would you say your health is excellent, very good, good, fair, or poor?
- ☐ Excellent
- ☐ Very good
- ☐ Good
- ☐ Fair
- ☐ Poor
- ☐ Don’t Know/Refused

31. How would you describe your household's financial situation?
- ☐ Don't have enough to meet basic expenses
- ☐ Meet only your basic expenses
- ☐ Meet your basic expenses with a little left over for extras
- ☐ Live comfortably
- ☐ Don’t Know/Refused

32. In general, how confident are you that you have enough money or health insurance to pay for the usual medical costs that you require for the next 30 days?
- ☐ Not at all confident
- ☐ Not too confident
- ☐ Somewhat confident
- ☐ Very confident
- ☐ Prefer not to answer
- ☐ Don’t Know/Refused

© 2023 Dusetzina SB et al. *JAMA Network Open.*

33. Have you EVER been told by a doctor or other health professional that you had any of the following medical conditions?

	Yes	No	Refused	Don't know
Alzheimer's Disease / Dementia				
Anxiety				
Asthma				
Cancer				
Chronic Kidney Disease				
Chronic Obstructive Pulmonary Disease (COPD)				
Depression				
Diabetes/Sugar Diabetes				
Glaucoma				
Heart Disease / Ischemic Heart Disease				
High Blood Pressure (Hypertension)				
High Cholesterol				
Irregular Heartbeat (Atrial Fibrillation)				
Osteoporosis				
Rheumatoid Arthritis / Osteoarthritis				

34. How often do you need to have someone help you when you read instructions, pamphlets, or other written material from your doctor or pharmacy?

- ☐ Always
- ☐ Often
- ☐ Sometimes
- ☐ Rarely
- ☐ Never
- ☐ Don't Know/Refused

35. Thank you for your time today! Your participation will help add valuable information to the development of medication cost discussion tools. Is there anything else that you would like to tell us?

Open text option.

© 2023 Dusetzina SB et al. *JAMA Network Open.*
