# Supplementary material for: Cost-Related Medication Nonadherence and Desire for Medication Cost Information Among Adults Aged 65 Years and Older in the US in 2022
Source: JAMA Netw Open. 2023 May 18;6(5):e2314211. doi: 10.1001/jamanetworkopen.2023.14211 (PMC10196872; doi:10.1001/jamanetworkopen.2023.14211)
Supplement: Supplement 2. — Data Sharing Statement [file jamanetwopen-e2314211-s002.pdf]

Data Sharing Statement

Dusetzina. Cost-Related Medication Nonadherence and Desire for Medication Cost Information Among Adults Aged 65 Years and Older in the US in 2022. *JAMA Netw Open*. Published May 18, 2023. doi:10.1001/jamanetworkopen.2023.14211

Data
Data available: No

Additional Information
Explanation for why data not available: We will be making the full survey questionnaire available via the online supplement.
